# Hepatic Encephalopathy and Treatment Modalities: A Review Article

**DOI:** 10.7759/cureus.28016

**Published:** 2022-08-14

**Authors:** Kapil Sharma, Shivani Akre, Swarupa Chakole, Mayur B Wanjari

**Affiliations:** 1 Medicine, Jawaharlal Nehru Medical College, Datta Meghe Institute of Medical Sciences, Wardha, IND; 2 Community Medicine, Jawaharlal Nehru Medical College, Datta Meghe Institute of Medical Sciences, Wardha, IND; 3 Research and Development, Jawaharlal Nehru Medical College, Datta Meghe Institute of Medical Sciences, Wardha, IND

**Keywords:** antioxidant, rifaximin, lactulose, ammonia, encephalopathy

## Abstract

Hepatic encephalopathy (HE) is a condition that is commonly seen in individuals suffering from liver cirrhosis. After excluding brain illness, HE is described as a range of neuropsychiatric disorders in individuals with liver impairment. It is characterized by personality changes, intellectual impairment, and a depressed level of consciousness. Toxins that are typically eliminated from the body by the liver build up in the blood and eventually reach the brain, causing HE. Many signs and symptoms of HE may often be treated if caught early and treated properly. It is important to remember that not everyone who is affected may experience every symptom mentioned below. Affected individuals should speak with their doctor and medical staff about their specific disease, associated symptoms, and general prognosis. Many people only have minor symptoms, known as minimal HE. The exact pathophysiology of HE is still being debated, with the primary theories focusing on neurotoxins, reduced neurotransmission caused by alterations in brain energy metabolism, systemic inflammatory response, and blood-brain barrier (BBB) disturbances in liver failure, as well as metabolic irregularities.

## Introduction and background

Hepatic encephalopathy (HE), which can manifest as many different neurological or mental diseases, from asymptomatic to coma, is a generic term for brain dysfunction brought on by hepatic insufficiency and/or portal-systemic shunting [1]. The underlying cause of liver illness is not taken into account in this definition of HE. However, chronic liver diseases (CLDs), including viral hepatitis, primary biliary cholangitis, alcohol-related liver disease, and non-alcoholic fatty liver disease, can all harm the brain through mechanisms unrelated to liver loss or dysfunction. A liver transplant (LT) is considered to be able to reverse the metabolic disorder known as HE. However, several studies have demonstrated that HE is characterized by neuroinflammation and neuronal cell death, and that extended durations of overt HE might have irreversible effects. These manifest as ongoing neurological issues following LT [2]. More significantly, regardless of the severity of the liver disease, HE has been related to a high risk of mortality, suggesting that it is a sign of hepatic insufficiency, but it may also have distinct implications for pathophysiology and prognosis [3].

## Review

Pathogenesis and causes

**Figure 1:**
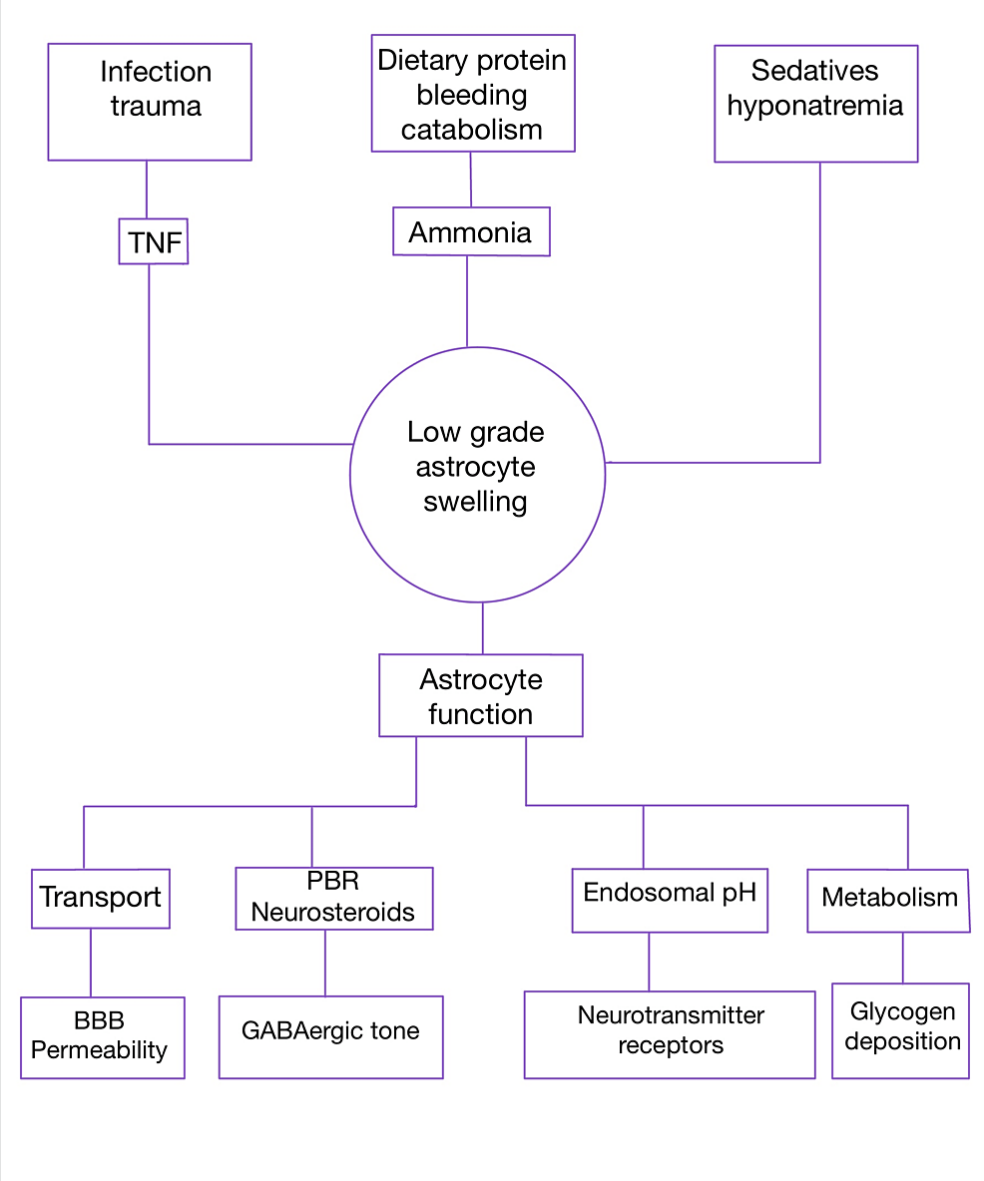
A hypothesis on how different precipitating causes could cause HE. According to this concept, low grade cerebral edema, which affects astrocyte hydration, is a critical event and one of the main mechanisms that causes astrocyte dysfunction and the clinical symptoms of HE. HE, hepatic encephalopathy

According to data from recent studies, ammonia continues to play a significant role in the pathophysiology of HE [4]. In most cases, ammonia is changed in the liver to urea and eliminated through the urine. The brain is severely harmed by ammonia. Despite the fact that ammonia is considered to play a part in the development of HE, some individuals with high ammonia levels may not exhibit symptoms, indicating that additional factors may be at play. Inadequate functioning of central nervous system cells known as astrocytes which help in maintaining the blood-brain barrier (BBB), causes dysfunction of the BBB, leading to the entry of harmful substances into the brain parenchyma causing permanent damage to it. To identify the precise underlying processes that cause HE and the symptoms that go along with it, more research is required. HE can be brought on by low oxygen levels in the body, the use of specific medications, especially those that have an impact on the central nervous system, such as benzodiazepines and other sleeping aids, antidepressants, and antipsychotics, as well as desiccation, decreased bowel movements, gastrointestinal bleeding, binge drinking, septicemia, renal irregularities, and more. HE can be triggered by surgery in rare situations. Gastrointestinal bleeding is the most frequent trigger associated with HE, most likely because this condition is more common in patients with cirrhosis than in the general population (Figure 1).

Ammonia

The neurotoxic ammonia is the most well studied in relation to HE. The gut produces ammonia as a byproduct of bacterial urease activity, protein digestion, and amino acid deamination. As a result, the concentration of ammonia in the systemic circulation is regulated by a functioning urea cycle in a healthy liver [5]. When there is any liver pathology leading to decreased functioning of the liver, it causes ammonia to build up in the system and thus leads to encephalopathy. Ammonia levels affect prognosis and are a crucial part of the development of HE and are a treatment target. In individuals with cirrhosis, astrocyte swelling brought on by hyperammonemia may be a crucial development factor for HE [6]. The conversion of ammonia to glutamine by astrocytes, which results in an increase in intracellular osmolarity, may be one cause of brain edema. When acute liver disease is evaluated biochemically or using postmortem material, brain glutamine contents are much higher [7].

Dysfunction in neurotransmission

Neuronal disinhibition is caused by altering neurotransmission systems Recent research suggests that the development of HE may be influenced by a dysfunctional lymphatic system, which aids in the elimination of different chemicals that build up in the brain [8].

Oxidative stress and inflammation

A damaged BBB and increased neuroinflammation are caused by the diseased liver, which also exacerbates systemic inflammation. Additionally, it encourages superimposed infection and gut bacterial translocation. Oxidative stress, a generalized condition that commonly exists in cirrhosis, may have an impact on BBB porosity because lipids, proteins, and DNA are highly reactive with reactive oxygen (and nitrogen) species [9] as well as minor weight loss. Different therapeutic alternatives for metformin-intolerant women with polycystic ovary syndrome (PCOS) should be examined as a result of metformin intolerance and its associated adverse effects.

Bile acids

Recently, it was shown that cirrhotic individuals with HE's cerebrospinal fluid (CSF) had enormous quantities of bile acids [10]. Regional cerebral edema in rats with acute galactosamine-induced liver failure has been demonstrated in animal models, proving that the BBB has lost its barrier function, at least in part [11].

Buildup of manganese

With high plasma levels brought on by the liver's inability to eliminate it, manganese has also been linked to the etiology of HE and has been found to accumulate in the basal ganglia. The correlation between this and pallidal signal hyperintensity has been established seen in cirrhotic patients' MRIs [12].

Inflammation

It is critical to emphasize that brain cell destruction contributes to the development of HE as well as being one of its side effects. It has been demonstrated that under these conditions, astroglia produces tumor necrosis factor (TNF)-, followed by the release of glutamate and the activation of microglia. The proliferation of microglia and the production of pro-inflammatory cytokines including TNF-, interleukin-1 (IL-1), and interleukin-6 (IL-6) are often observed after microglia activation [13]. Animal and human research has provided evidence that elevated ammonia only causes HE when there is a systemic inflammatory response syndrome (SIRS) [14]. Thus, it is generally agreed that altered nitrogen metabolism caused by sepsis, as well as the release of pro-inflammatory mediators, might cause HE in cirrhotic patients [15].

Symptoms and signs

**Figure 2:**
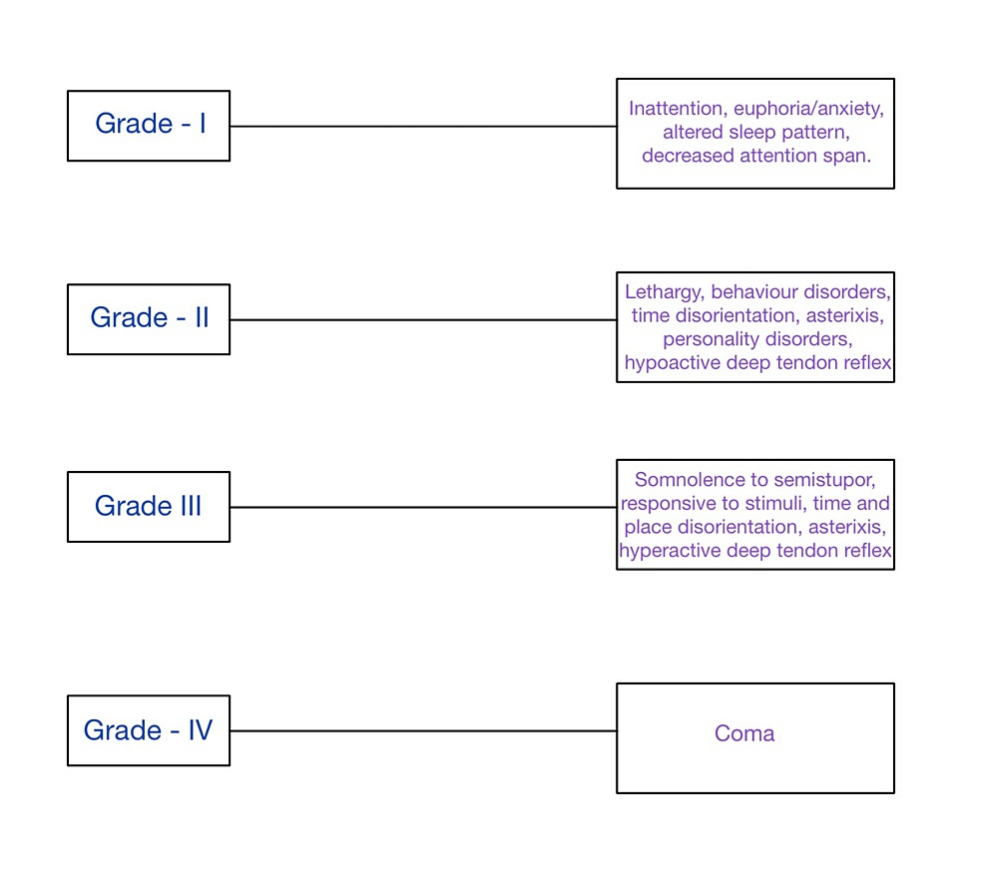
Grades of HE (West Haven criteria). HE, hepatic encephalopathy

A wide range of generalized neurological and psychological symptoms are brought on by HE. The signs of brain dysfunction include poor mental clarity and confusion. In the early stages, modest changes in behavior, demeanor, and logical reasoning can be seen. The person's attitude might shift, and their judgment could be clouded. Possible disruptions might occur to regular sleep habits. People could experience anxiety, depression, or irritability. They can struggle to focus. The individual may have a musty, sweet breath smell at any stage of encephalopathy. One of the signs of mild HE is musty or sweet breath, along with analytical difficulties, personality changes, poor focus, difficulties writing or losing other tiny hand movements, disorientation, amnesia, and poor judgment. Confusion, sleepiness or lethargy, anxiety, convulsions, profound personality changes, exhaustion, incoherent speech, trembling hands, and sluggish movements are some symptoms of severe HE. As the condition worsens, patients find it difficult to maintain their hands firm when they extend their arms, which causes their hands to flail around crudely (asterixis). People may jerk their muscles unintentionally or after being subjected to a sudden noise, light, movement, or other stimuli. The name for this jerky is myoclonus. Additionally, patients frequently experience confusion and drowsiness, as well as slow motions and speech. It is typical to feel disoriented. Encephalopathy patients seldom feel angry or aroused. They may eventually go unconscious and into a coma as their liver function continues to decline. Despite therapy, coma frequently results in death (Figure 2) [16].

Treatment modalities in patients with hepatic encephalopathy

Depending on how severe HE is, several therapy modalities are used. The goal is to maximize the body's ability to remove ammonia from the bloodstream while reducing ammonia production, which remains the major priority. However, ammonia metabolism is intricate and controlled by several organs, including the brain, muscles, kidneys, and liver. In order to optimize their efficacy, medications used to treat HE must be well studied and put through controlled clinical studies. A significant barrier to effective therapy for HE is the absence of interventions for other triggering variables such as oxidative stress, inflammation, or other brain changes. Managing the consequences of encephalopathy, while actively detecting the underlying cause, is crucial. The first step in therapy is to address any potential precipitating causes, such as infections, electrolyte imbalances, dehydration, etc. after the patient is well after the initial episode, it is time to address the recurrence of HE. Even though mild and episodic HE are minor in nature, they can significantly impact a patient's ability to live a normal life. Sadly, only overt hepatic encephalopathy (OHE) is currently regularly treated, and there are few other medical alternatives for treating HE as a whole. Finally, a more individualized course of therapy will need to be created for the patients, taking into account not only the stage of HE but also the underlying disease and its past.

Non-absorbable Disaccharides and Polyethylene Glycol

Standard therapies that try to lessen the quantity of ammonia absorbed into the bloodstream include lactulose and, to a lesser extent, lactitol. Lactulose has several effects, including acting as a laxative and producing a hyperosmolar environment that hinders the colon's ability to effectively absorb ammonia. Despite the paucity of research supporting the use of lactulose in the treatment of acute HE, a recent meta-analysis demonstrated the efficacy of non-absorbable disaccharides in the management and prevention of HE. Other advantages include a decrease in fatalities from all causes and significant liver-related morbidities [17]. It also demonstrated that non-absorbable disaccharides therapy might lessen major liver disease-related side effects such as liver failure, hepatorenal syndrome, and variceal hemorrhage [18].

Antibiotics

There has been studies on oral antibiotics being used to regulate intestinal flora and lessen ammonia generation as a treatment for HE. Neomycin, an aminoglycoside antibiotic, helps to inhibit glutaminase and reduce ammonia levels while being poorly absorbed and reaching large amounts in the stomach [19]. The use of antibiotics in HE was first made popular by this substance. Neomycin's usage is currently not permitted in clinical practice. A semi-synthetic, non-absorbable antibiotic derived from rifamycin is known as rifaximin. When it comes to lowering blood ammonia levels, rifaximin has been demonstrated to be at least as effective as neomycin with fewer side effects [20]. It has anti-inflammatory qualities and exerts its effects via altering the makeup and metabolism of the gut microbiota, among other ways [21]. It has been demonstrated that rifaximin and lactulose combined treatment is more successful than rifaximin alone [22].

Probiotics

Live bacteria supplements known as probiotics are thought to help intestinal dysbiosis and reduce ammonia output. Probiotic therapy may help in the onset of overt HE and reduce the plasma ammonia levels, according to a Cochrane systematic review, but it has minimal impact on mortality [18].

L-Ornithine L-Aspartate (LOLA)

The production of glutamine and the urea cycle, two crucial processes during ammonia detoxification, are stimulated when L-ornithine L-aspartate (LOLA) is used as a supplement [23]. Comparatively to placebo or no-intervention controls, findings based on meta-analyses show that in cirrhotic individuals with covert hepatic encephalopathy (CHE) and HE, L-ornithine-L-aspartate is superior at symptom recovery and blood ammonia level reduction [23].

Intravenous Albumin Infusion

After studies demonstrated that it improves outcomes for those having cirrhosis with hepatorenal syndrome or spontaneous bacterial peritonitis, it became widely used in patients with the disease. The alleviation of oxidative stress reduction and plasma expansion to cause vascular dysfunction is hypothesized to be the mechanism of action [23].

Nutritional Supplement

Branched chain amino acids (BCAA) such as valine, leucine, isoleucine, etc, are commonly deficient in patients with liver cirrhosis. Skeletal muscles also help in the detoxification of ammonia with the amidation process for glutamine synthesis using BCAAs. So, it is essential to give BCAA supplements to patients with liver cirrhosis.

Antioxidant Supplement

These help in improving the general health of patients, reduce oxidative stress on the body, especially the liver, and help in faster regeneration. Proper nutrition and antioxidants help reduce the chances of carcinoma and other complications related to the hepatic system [20].

## Conclusions

In individuals with cirrhosis associated with end-stage liver disease, HE plays a substantial role in morbidity. The unexpected nature of HE has a significant negative influence on patients and reduces their quality of life. First, ask the patient to avoid further alcohol consumption and start lactulose solution as soon as possible to reduce the blood ammonia levels and prevent further irreversible brain parenchymal damage. New and forthcoming therapeutic options have been created as a result of research into the complexity of HE. The mainstays of therapy continue to be avoiding HE precipitants and a mixture of lactulose and rifaximin. For acute increase in blood ammonia levels, LOLA should be prescribed as it led to a rapid decrease in blood ammonia levels. Nutrition and antioxidant supplement should be continued for the general health of patients and to prevent further progress of cirrhosis into carcinoma. The main aim in patients with HE is to reduce the blood ammonia levels to prevent brain parenchymal damage which can lead to permanent mental abnormalities. Future research should focus on identifying further innovative pathways and therapeutic targets in the hopes of really helping people with HE.
